# Landmark-based early perfusion phenotyping for prognostic stratification in septic shock: derivation in MIMIC-IV and fixed-parameter external transportability assessment in eICU

**DOI:** 10.3389/fmed.2026.1880921

**Published:** 2026-07-28

**Authors:** Shuo Yang, Zhihao Ren, Yiran Li, Zihang Zhao, Changsheng Sun, Lin Liu, Lin Jin, Ling Wang, Zhiyong Hou

**Affiliations:** 1 Department of Orthopaedic Surgery, The Third Hospital of Hebei Medical University, Shijiazhuang, Hebei, China; 2 Orthopaedic Research Institute of Hebei Province, Shijiazhuang, Hebei, China; 3 Department of Orthopedic Oncology, The Third Hospital of Hebei Medical University, Shijiazhuang, Hebei, China; 4 NHC Key Laboratory of Intelligent Orthopaedic Equipment (The Third Hospital of Hebei Medical University), Shijiazhuang, Hebei, China; 5 Engineering Research Center of Orthopedic Minimally Invasive Intelligent Equipment, China Ministry of Education, Shijiazhuang, Hebei, China; 6 Key Laboratory of Biomechanics of Hebei Province, Shijiazhuang, Hebei, China; 7 Key Laboratory of Precise Assessment, Diagnosis, and Treatment of Soft Tissue Injury of Hebei Province, Shijiazhuang, Hebei, China

**Keywords:** critical care databases, lactate clearance, perfusion, prognostic stratification, septic shock, urine output

## Abstract

**Background:**

Septic shock is clinically heterogeneous, and early perfusion failure may not be adequately captured by any single bedside variable. We aimed to derive a clinically interpretable early perfusion phenotyping framework within a landmark design and assess whether the high-risk phenotype contour could be transferred across critical care databases.

**Methods:**

In this retrospective observational study, MIMIC-IV was used for phenotype derivation and the eICU Collaborative Research Database for fixed-parameter external transportability assessment. Adults in the first intensive care unit stay were eligible if they had early vasopressor exposure and baseline lactate greater than 2 mmol/L. At an 8-h landmark, phenotypes were derived using mean arterial pressure deficit burden, body-weight-standardized urine output, and lactate clearance. The primary outcome was in-hospital mortality, selected for cross-database harmonization because it was consistently available in both databases, whereas fixed-time 28- or 90-day mortality was not reliably harmonized. Secondary renal outcomes were summarized descriptively.

**Results:**

The MIMIC landmark cohort included 4,374 patients, of whom 2,671 had complete data for phenotype assignment. Four early perfusion phenotypes were identified. Phenotype 1 was characterized by greater hypotensive burden, poorer lactate clearance, and lower urine output. In-hospital mortality was 47.0% in Phenotype 1 versus 17.7% in Phenotype 4. Phenotype 1 remained associated with higher mortality after primary adjustment for age, sex, and pre-landmark SOFA score (OR 3.67, 95% CI 2.74–4.93), but this estimate attenuated after expanded pre-landmark case-mix adjustment (OR 2.56, 95% CI 1.85–3.53). In eICU, the same high-risk contour remained detectable under frozen transfer among measured complete-case patients. However, the strict and relaxed transfer cohorts were small, monitoring-dense subsets, and class-prevalence drift with measurement-dependent complete-case formation precluded robust conclusions about transportability of the entire four-class model, particularly for Phenotypes 2–4.

**Conclusions:**

A landmark-based early perfusion phenotype identified a high-risk perfusion-failure contour among septic shock patients with sufficient early monitoring density. In eICU, this contour was recoverable only within measured complete-case subsets, and the external data do not establish robust transportability of the entire four-class model. The framework should be interpreted as an observational, hypothesis-generating prognostic stratification approach rather than as an independently validated bedside decision tool.

## Introduction

Sepsis and septic shock remain leading causes of critical illness and death worldwide despite advances in antimicrobial therapy, source control, organ support, and hemodynamic resuscitation (1–5). Yet patients who meet contemporary septic shock criteria represent a clinically heterogeneous population, with substantial variation in the severity and configuration of circulatory failure, metabolic derangement, renal vulnerability, treatment requirements, and outcomes (2–5). This heterogeneity limits the value of syndrome-level labels alone, which are often insufficient for individualized risk stratification, pathophysiologic characterization, and clinical trial enrichment.

Clinical subphenotyping has consequently emerged as a central strategy for addressing heterogeneity in sepsis research. Prior studies have shown that data-driven phenotypes can recover clinically meaningful variation within broadly defined sepsis syndromes and identify groups with different outcome profiles and possible therapeutic relevance (6, 7). However, many existing frameworks rely on broad combinations of demographic, laboratory, organ dysfunction, and trajectory variables, which may improve statistical separation at the cost of physiologic interpretability. For studies aimed at early ICU risk stratification, a narrower construct anchored in a single clinically relevant pathophysiologic domain may therefore be better suited.

A particularly relevant construct in this context is early perfusion failure. In septic shock, impaired tissue perfusion is not captured by any single definitive bedside measure, but rather by multiple partially complementary signals. MAP reflects exposure to insufficient macrohemodynamic pressure support, and cumulative hypotensive burden has been associated with adverse outcomes and kidney injury (8, 9). Lactate clearance remains a pragmatic marker of early resuscitation response and is widely used in sepsis care (10, 11). Urine output provides an immediately available integrative signal of kidney response to circulatory stress and systemic illness and is strongly associated with AKI and mortality in critically ill patients (12, 13). Taken together, these variables may offer a compact and clinically interpretable representation of an evolving early perfusion state.

A second methodological challenge in observational phenotype research is temporal alignment. If phenotype-defining variables accrue over the first several hours after ICU admission while outcome follow-up begins at time zero, the analysis becomes vulnerable to time-related bias, including misalignment between exposure ascertainment and risk attribution, as well as immortal-time-type problems (14, 15). This issue is especially relevant for repeat lactate testing, which is clinically triggered rather than randomly observed. A landmark framework provides a transparent solution by restricting phenotype assignment to information available up to a prespecified time point and initiating follow-up only after phenotype definition is complete (14, 15).

We therefore aimed to derive early perfusion-related phenotypes in MIMIC-IV using a parsimonious set of routinely available bedside perfusion variables within a strict 8-h landmark framework, and then to assess fixed-parameter external transportability of phenotype contours in eICU using MIMIC-derived preprocessing constants and recovered model parameters rather than external refitting. We hypothesized that this approach would identify a clinically coherent high-risk contour characterized by persistent early perfusion dysfunction, while recognizing that external database transfer may preserve overall risk layering without reproducing every latent class distribution.

## Materials and methods

### Study design and reporting

This was a retrospective observational study reported in accordance with the STROBE statement (16). The analysis was designed as a prognostic stratification and association study, not as a causal inference study and not as a deployable clinical prediction tool. MIMIC-IV served as the derivation cohort, whereas the eICU Collaborative Research Database served as the fixed-parameter external transportability cohort.

### Data sources and ethical considerations

MIMIC-IV is a large, deidentified single-center critical care database derived from Beth Israel Deaconess Medical Center (17). The eICU Collaborative Research Database is a deidentified multicenter ICU database spanning hospitals across the United States (18). Both databases are publicly available to credentialed investigators under data use agreements, and the source database publications describe institutional review board oversight and waiver of informed consent for release of deidentified data (17, 18). Because the present study was a secondary analysis of fully deidentified public data, additional informed consent was not required. The Ethics Committee of the Third Hospital of Hebei Medical University determined that analysis of these fully deidentified public data was exempt from institutional ethics review.

### Cohort roles and cross-database harmonization

We used a prespecified asymmetric derivation-transfer design. MIMIC-IV served as the sole derivation cohort for phenotype development. The eICU Collaborative Research Database was used exclusively for fixed-parameter external transportability assessment, in which MIMIC-derived preprocessing constants and recovered mixture parameters were applied without external refitting. Accordingly, the eICU analysis was designed to examine whether the high-risk perfusion contour, assignment certainty, class-prevalence pattern, and crude risk layering were recoverable in another database. It was not designed or reported as independent external validation of all latent classes or as clinical validation of a bedside decision tool. For interpretive purposes, the strict 8-h structure was treated as the primary transportability analysis, whereas the relaxed 12-h structure was prespecified as a sensitivity transportability analysis introduced to accommodate lower repeat-lactate availability in eICU.

Case identification was harmonized as closely as possible but was not identical across databases. In MIMIC, eligible patients were identified within a Sepsis-3 first-ICU cohort (2–4). In eICU, because complete Sepsis-3 reconstruction was not used in the prespecified transportability framework, a sepsis-related diagnosis anchor served as the database-constrained entry criterion. In both databases, the operational shock definition required adult age, first ICU stay, vasopressor exposure within 6 h of ICU admission, baseline lactate > 2 mmol/L, and application of the same prespecified landmark structure and phenotype-defining feature windows. Because cohort entry definitions and measurement processes differed across databases, between-database class-prevalence comparisons were treated as descriptive transportability summaries rather than as estimates of true population-level phenotype prevalence differences.

### Study population

#### MIMIC derivation cohort

The MIMIC source population comprised 94,458 ICU stays. After restriction to adult first ICU admissions, the analytic pipeline identified 27,881 Sepsis-3 first-ICU stays, of whom 9,781 received vasopressors within 6 h and 4,449 met the operational septic shock candidate definition of baseline lactate > 2 mmol/L. The final 8-h landmark cohort included 4,374 patients. Within this cohort, MAP deficit burden and standardized urine output were available for nearly all patients, whereas complete-case formation was driven primarily by availability of repeat lactate measurement in the prespecified 4–8 h window. The final complete-case derivation cohort included 2,671 patients with all three phenotype-defining features available, and all derivation analyses were performed in this analytic set.

#### eICU fixed-parameter external transportability cohort

In eICU, the source population comprised 157,883 adult first ICU stays. The upstream operational cohort was defined by intersection of three prespecified criteria rather than a sequential screening cascade: a sepsis-related diagnosis anchor, vasopressor exposure within 6 h of ICU admission, and baseline lactate > 2 mmol/L, yielding 2,277 patients in the operational septic shock candidate cohort. Two external transportability assessment structures were prespecified: a strict 8-h structure that mirrored the derivation framework as closely as possible, and a relaxed 12-h structure introduced to accommodate lower repeat-lactate availability in eICU while preserving a clinically plausible early reassessment window. These structures yielded landmark cohorts of 2,163 and 2,090 patients, respectively. In both structures, MAP was nearly always recoverable, whereas complete-case formation was driven primarily by urine output and repeat lactate availability. The final complete-case cohorts available for frozen phenotype assignment included 432 patients from 60 hospitals in the strict structure and 666 patients from 71 hospitals in the relaxed structure.

### Landmark framework and feature definitions

A central feature of the analytic design was temporal alignment between phenotype ascertainment and outcome follow-up. In MIMIC, the landmark was fixed at 8 h after ICU admission. All phenotype-defining variables were fully ascertained by that landmark, and all follow-up began only thereafter. In eICU, the same principle was preserved through two prespecified alignment structures: a strict 8-h structure and a relaxed 12-h structure. For each eICU transfer structure, feature availability was assessed within the corresponding landmark-eligible cohort. MAP deficit burden and standardized urine output retained the same 0–8 h definitions in both eICU structures, whereas the repeat-lactate reassessment window was 4–8 h in the strict structure and 4–12 h in the relaxed structure. The relaxed structure was introduced to accommodate lower repeat-lactate availability in eICU while preserving the core requirement that phenotype-defining information be observed before follow-up began.

The three phenotype-defining variables were as follows. First, 0–8 h MAP deficit burden was defined using a target MAP threshold of 65 mmHg. For each observed segment, MAP deficit was calculated as max (0, 65–MAP), and the area under the MAP-deficit curve was obtained by summing deficit × segment-duration terms. MAP deficit burden was then calculated as the AUC divided by total covered MAP observation time within the 0–8 h window. Second, 0–8 h body-weight-standardized urine output was calculated as total urine volume recorded during the 0–8 h window divided by body weight and by 8 h, yielding a standardized rate in mL/kg/h. Body weight was defined using the first available ICU weight recorded at or before the 8-h landmark according to a prespecified rule; when multiple eligible weights were available, the earliest qualifying measurement was used. If no eligible pre-landmark ICU weight was available, standardized urine output was treated as unavailable for phenotype construction. Urine output was aggregated from prespecified volume-based ICU output records before the landmark after exclusion of implausible values. Third, baseline lactate was defined as the blood lactate value closest to ICU admission within a window extending from 6 h before to 6 h after ICU admission. Lactate clearance was calculated as the percentage change from baseline lactate to a repeat lactate value obtained within the prespecified early reassessment window (4–8 h in MIMIC and strict eICU; 4–12 h in relaxed eICU). When multiple repeat lactate measurements were available within the reassessment window, the measurement closest to the landmark time was selected according to the archived extraction rule.

Before model fitting, each feature underwent winsorization at the 1st and 99th percentiles and was then standardized to a z score using the post-winsorization mean and standard deviation. In eICU, frozen preprocessing constants derived from MIMIC were applied without recalibration. Complete availability of all three phenotype-defining features was required for phenotype derivation in MIMIC and for frozen phenotype assignment in eICU. No imputation was performed.

### Phenotype derivation and class-number selection

Phenotypes were derived in MIMIC using a four-class Gaussian mixture model with diagonal covariance structure based on the three standardized phenotype-defining features (19). Candidate solutions with 2 to 6 classes were evaluated using a prespecified descriptive comparison framework rather than information criteria alone. Operationally, we considered AIC and BIC, minimum class proportion, assignment certainty as reflected by mean maximum posterior probability, resampling stability, and clinical interpretability of the resulting feature profiles. Because information criteria continued to improve with higher-class solutions, the retained four-class solution was not interpreted as the numerically optimal density model. Instead, it was selected as a pragmatic, parsimonious working partition that maintained acceptable class size, assignment certainty, stability, and interpretability while avoiding increasingly fragmented higher-class solutions. The high-risk profile was described after retention of the four-class solution and was not treated as an objective class-number selection criterion. Final class assignment was based on maximum posterior probability. For reporting purposes, the retained classes were labeled neutrally as Phenotype 1 through Phenotype 4, and Phenotype 4 was used as the reference category in downstream association analyses.

### Resampling stability and fixed-parameter external transportability assessment

Model stability was evaluated using 30 bootstrap repetitions and 20 split-sample repetitions. Concordance between each repeated solution and the primary phenotype solution was summarized using the adjusted Rand index (ARI) (20). These analyses were used to assess whether the retained phenotype structure was reproducible under resampling perturbations rather than being driven by a single sample realization.

Fixed-parameter external transportability assessment was conducted without external refitting. The MIMIC 8-h winsorization cut points, post-winsorization means and standard deviations, and the recovered four-class diagonal GMM parameter set (including class weights and class-specific means and variances for each standardized feature) were applied directly to eICU. Accordingly, the eICU objectives were limited to assessment of phenotype-contour recovery, class-prevalence pattern, assignment certainty, and crude risk layering. The eICU analysis was not intended to reproduce adjusted mortality associations from the derivation cohort and should not be interpreted as independent external clinical validation.

### Outcomes and covariates

The primary outcome was in-hospital mortality. Secondary renal outcomes were 72-h AKI onset/progression and 72-h incident RRT. Because body-weight-standardized urine output was one of the phenotype-defining variables, renal outcomes were prespecified as descriptive secondary summaries rather than as independent validation endpoints. To reduce direct definitional overlap between the urine-output-based phenotype variable and AKI ascertainment, the primary AKI analysis used a creatinine + CRRT-based route rather than reintroducing urine output into the AKI definition (21). However, this approach does not eliminate physiologic coupling between early oliguria, perfusion failure, AKI progression, and RRT. Because the archived renal summaries used total phenotype counts as denominators, secondary renal outcomes are reported only as descriptive phenotype-level event proportions rather than as adjusted associations or formal risk-set incidence estimates.

The prespecified adjusted mortality model included age, sex, and pre-landmark SOFA score (22). All SOFA components were restricted to information available before the landmark. First-24-h SOFA was not used because it would incorporate post-landmark information into the adjustment set.

### Sensitivity and supplementary analyses

Several prespecified analyses were performed to assess robustness and clarify interpretation boundaries. First, class-number comparison was conducted across candidate solutions with 2 to 6 classes. Second, included-versus-excluded analyses were used to characterize the selection pattern associated with complete-case formation in each database. Third, inverse probability weighting (IPW) was used in MIMIC as a sensitivity analysis to address non-random selection into the complete-case derivation cohort. The weighting model incorporated baseline demographic and severity variables available at cohort entry, together with baseline lactate and ICU admission unit, and was used to examine whether the primary mortality association was materially altered after reweighting. Fourth, incremental-value analyses compared apparent predictive performance across a clinical base model (age + sex + SOFA), the base model plus phenotype, and the base model plus the three continuous phenotype-defining variables. Fifth, an expanded adjusted sensitivity analysis for in-hospital mortality was performed in MIMIC to examine robustness to broader pre-landmark case-mix adjustment. In addition to age, sex, and pre-landmark SOFA, the expanded model incorporated baseline lactate, grouped first ICU care unit, pre-landmark invasive ventilation status, and pre-landmark RRT, while avoiding re-entry of the phenotype-defining variables themselves. These analyses were interpreted as descriptive assessments of added prognostic information rather than evidence of bedside clinical utility. The need for future sensitivity analyses using constrained or non-Gaussian clustering models is acknowledged as a limitation.

### Statistical analysis

Categorical variables are reported as counts and percentages. Continuous baseline variables are reported as means with standard deviations when used for baseline description, while other archived analytic summaries are reported according to the prespecified final reporting format. In MIMIC, associations between phenotype membership and in-hospital mortality were estimated using multivariable logistic regression, with Phenotype 4 specified as the reference category. Secondary renal outcomes in MIMIC and fixed-parameter transportability outcomes in eICU were summarized descriptively according to the prespecified analytic framework. All inferential tests were two-sided, with emphasis placed on effect sizes, confidence intervals, and internal consistency of the findings rather than isolated *P*-values. No attempt was made to estimate treatment effects, phenotype-specific treatment response, or causal mediation. Data extraction and cohort construction were performed using SQL queries, and downstream statistical analyses were performed using prespecified R and Python scripts.

## Results

### Cohort construction and analytic samples

The MIMIC 8-h landmark derivation cohort included 4,374 patients, of whom 2,671 (61.1%) had complete data for MAP deficit burden, standardized urine output, and repeat-lactate-derived clearance and therefore entered phenotype derivation (Table 1). Availability differed substantially across the three phenotype-defining features: MAP deficit burden was available for 4,373 patients, standardized urine output for 4,186, and repeat-lactate-derived clearance for 2,811, indicating that repeat-lactate availability was the principal bottleneck to complete-case formation. In eICU, the upstream operational septic shock candidate cohort consisted of 2,277 patients meeting the intersecting criteria of a sepsis-related diagnosis anchor, vasopressor exposure within 6 h of ICU admission, and baseline lactate > 2 mmol/L (Table 2A). The prespecified strict 8-h and relaxed 12-h transfer structures yielded landmark cohorts of 2,163 and 2,090 patients, respectively. Within these landmark cohorts, MAP deficit burden was available in 2,152 and 2,079 patients, standardized urine output in 1,054 and 1,027 patients, and repeat-lactate-derived clearance in 847 and 1,264 patients, respectively. Complete availability of all three phenotype-defining variables was present in 432 patients from 60 hospitals in the strict structure and 666 patients from 71 hospitals in the relaxed structure, and these patients entered frozen phenotype assignment (Table 2B). Thus, the eICU transfer cohorts represented measured subsets of the operational cohorts, with complete-case formation driven primarily by urine output and repeat-lactate availability rather than by MAP availability.

**TABLE 1:** Construction of the MIMIC derivation cohort and complete-case analytic sample.

Step	Patients, *n*
All ICU stays	94,458
Adult first ICU stays	65,366
Sepsis-3 first ICU cohort	27,881
Vasopressor exposure within 6 h of ICU admission	9,781
Operational septic shock candidate cohort (baseline lactate >2 mmol/L)	4,449
8-h landmark cohort	4,374
0–8 h MAP deficit burden available	4,373
4–8 h lactate clearance available	2,811
0–8 h standardized urine output available	4,186
Complete-case phenotype derivation cohort	2,671

All downstream phenotype-based association analyses in MIMIC used the complete-case cohort with all three phenotype-defining features available.

**TABLE 2A:** Upstream operational cohort definition for fixed-parameter external transportability assessment in eICU.

Item	Patients, *n*	Note
Adult first ICU stays	157,883	Base fixed-parameter transfer assessment cohort
Sepsis-related diagnosis anchor	20,714	Marginal count
Vasopressor exposure within 6 h of ICU admission	12,616	Marginal count
Baseline lactate > 2 mmol/L	22,053	Baseline window: −6 h to +6 h
Intersection of all three criteria	2,277	Operational septic shock candidate cohort

**TABLE 2B:** Landmark reconstruction and complete-case analytic sample formation in eICU.

Step	Strict 8 h	Relaxed 12 h	Note
Operational septic shock candidate cohort	2,277	2,277	Upstream intersection cohort
Landmark cohort	2,163	2,090	Strict: 8 h; relaxed: 12 h
0–8 h MAP deficit burden available	2,152	2,079	Within-structure availability
0–8 h standardized urine output available	1,054	1,027	Within-structure availability
Repeat lactate available within window	847	1,264	Strict: 4–8 h; relaxed: 4–12 h
Complete-case cohort	432	666	Entered frozen assignment
No. of hospitals represented	60	71	Hospitals represented

Feature-availability counts are reported within the corresponding landmark cohort for each transfer structure. MAP deficit burden and standardized urine output were defined over the 0–8 h window in both structures, whereas repeat lactate was assessed over 4–8 h in the strict structure and 4–12 h in the relaxed structure. Overall crude in-hospital mortality in the complete-case cohorts was 34.26% in the strict structure and 33.33% in the relaxed structure.

Additional audit of urine-output standardization showed that no patient in either database was excluded from phenotype assignment solely because of missing eligible weight documentation when the other phenotype-defining variables were otherwise available. In MIMIC, 15 patients had recorded 0–8 h urine volume but unavailable body-weight-standardized urine output; however, none of these patients also had complete MAP deficit burden and lactate-clearance data. In eICU, no patient with recorded 0–8 h urine volume lacked eligible weight documentation or standardized urine-output calculation in either the strict or relaxed transfer structure.

**FIGURE 1:**
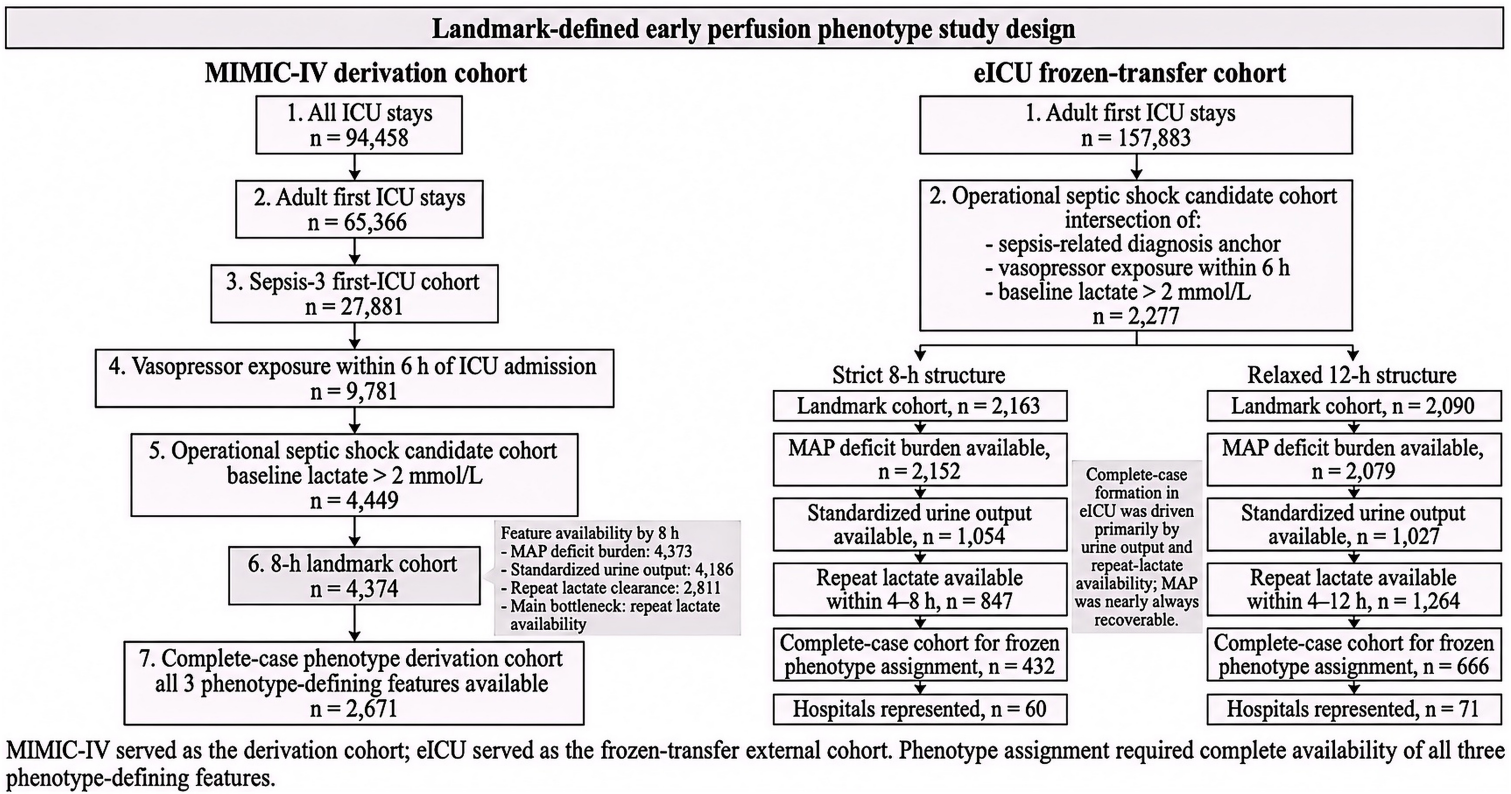
Cohort construction and complete-case sample formation in MIMIC-IV and eICU. MIMIC-IV served as the derivation cohort and eICU served as the fixed-parameter external transportability cohort. In both databases, phenotype derivation or assignment required complete availability of all three phenotype-defining features. In MIMIC-IV, complete-case formation was driven primarily by repeat-lactate availability. In eICU, feature availability was summarized within the corresponding landmark cohort for each transfer structure. MAP deficit burden and standardized urine output were defined over 0–8 h in both eICU structures, whereas repeat-lactate availability was assessed over 4–8 h in the strict structure and 4–12 h in the relaxed structure. Complete-case formation in eICU was driven primarily by urine output and repeat-lactate availability, whereas MAP was nearly always recoverable.

The overall derivation and eICU transportability cohort-construction pathways, together with complete-case sample formation in both databases, are summarized in Figure 1.

### Four early perfusion-related phenotypes in MIMIC

The retained 4-class solution identified four distinct early perfusion patterns (Table 3). Phenotype 1 comprised 606 patients (22.7%) and was characterized by higher MAP deficit burden (MAP z 1.243), poorer lactate clearance (lactate clearance z −0.518), and lower urine output (urine output z −0.604), a profile interpreted as a persistent perfusion-failure pattern. Phenotype 2 comprised 652 patients (24.4%) and showed lower MAP deficit burden with intermediate overall risk. Phenotype 3 comprised 961 patients (36.0%) and showed relatively lower MAP deficit burden and better lactate clearance but persistently lower urine output. Phenotype 4 comprised 452 patients (16.9%) and had the most favorable urine-output profile and the lowest crude risk.

**TABLE 3:** Distribution and feature profile of the four perfusion-related phenotypes in MIMIC.

Phenotype	*N*	Proportion, %	MAP deficit burden z	Lactate clearance z	Urine output z	Pre-landmark SOFA, mean
Phenotype 1	606	22.7	1.243	−0.518	−0.604	5.36
Phenotype 2	652	24.4	−0.684	0.132	0.177	4.90
Phenotype 3	961	36.0	−0.376	0.204	−0.374	5.00
Phenotype 4	452	16.9	0.121	0.069	1.350	4.36

Phenotype 1 was interpreted as a persistent perfusion-failure pattern; Phenotype 4 showed the most favorable crude risk profile. Feature values are empirical phenotype means of standardized z scores after 1st/99th percentile winsorization and subsequent standardization.

Retention of four classes was based on the class-number comparison summarized in Supplementary Table 1. Although higher-class solutions yielded further numerical improvement in AIC and BIC, the 5-class and 6-class solutions generated smaller and more fragmented partitions with lower assignment certainty than the retained 4-class solution. The 2-class and 3-class solutions provided coarser partitions that merged feature profiles that were more clearly separated in the 4-class solution. For a framework intended to support clinically interpretable stratification rather than maximal density estimation or individual-level prediction, the 4-class solution was retained as a pragmatic parsimonious representation with acceptable class size, assignment certainty, stability, and interpretability. The mortality-enriched Phenotype 1 profile was therefore interpreted as a feature of the retained solution rather than as a criterion used to select the number of classes.

**FIGURE 2:**
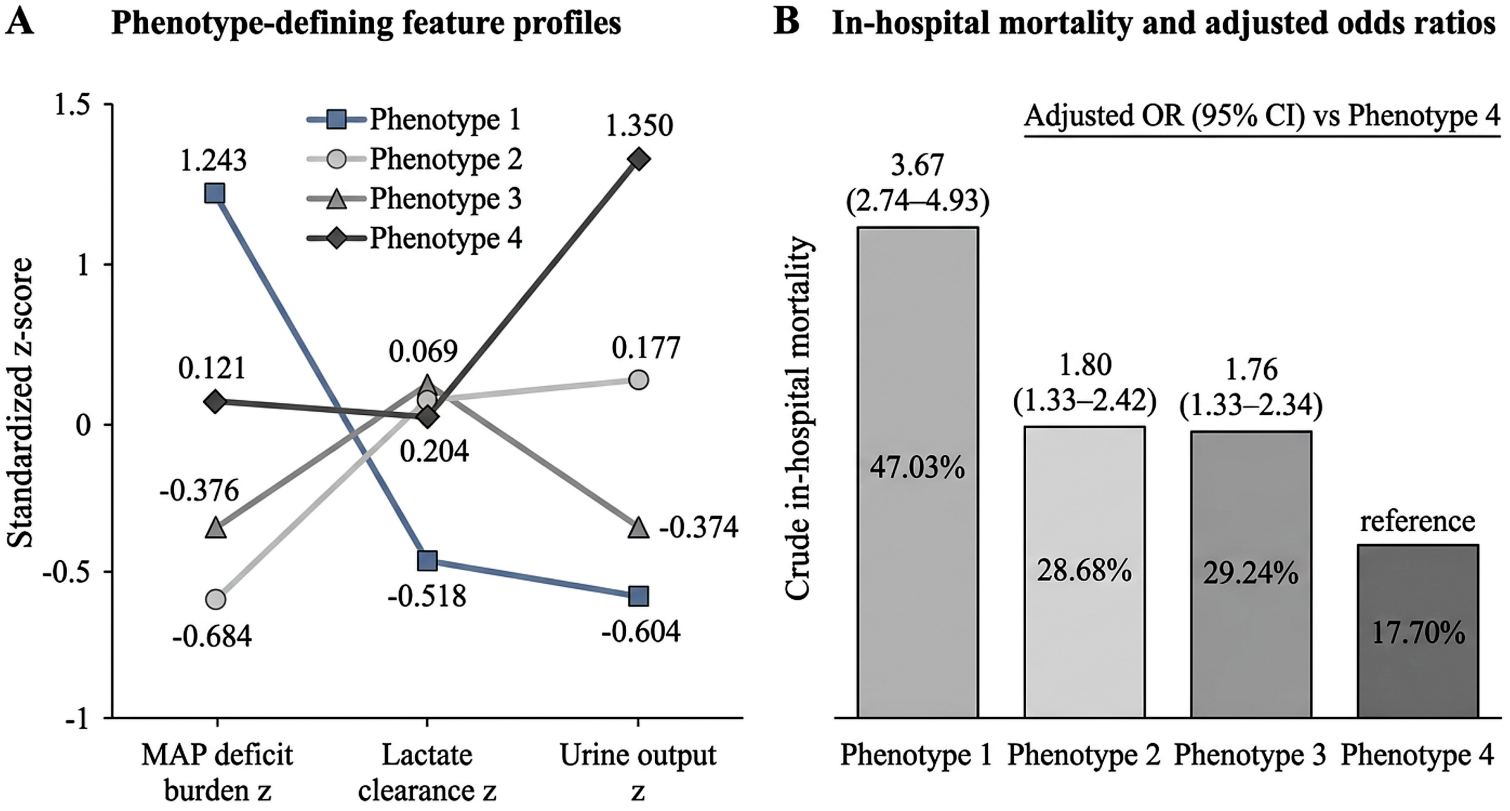
Early perfusion phenotype profiles and mortality layering in the MIMIC derivation cohort. Panel **(A)** shows the standardized phenotype-defining feature profiles for the four retained phenotypes in MIMIC-IV. Phenotype 1 was characterized by greater MAP-deficit burden, poorer lactate clearance, and lower urine output, consistent with a persistent early perfusion-failure pattern. Panel **(B)** shows crude in-hospital mortality and adjusted odds ratios for in-hospital mortality relative to Phenotype 4. Phenotype 1 showed the highest mortality risk, whereas Phenotype 4 showed the lowest crude risk profile.

The standardized feature profiles and corresponding mortality layering of the four retained phenotypes in the MIMIC derivation cohort are shown in Figure 2.

### Baseline characteristics in the derivation cohort

Baseline clinical characteristics across the four retained phenotypes are summarized in Table 4. In the MIMIC complete-case derivation cohort, mean age was 65.84 years, 41.33% were female, mean pre-landmark SOFA was 4.95, and mean baseline lactate was 4.76 mmol/L. Across phenotypes, Phenotype 1 showed the highest baseline severity markers, with the highest baseline lactate (5.49 mmol/L) and highest mean pre-landmark SOFA (5.36), whereas Phenotype 4 showed the lowest mean SOFA score (4.36) and lowest baseline lactate (4.06 mmol/L). These baseline gradients were directionally consistent with subsequent mortality separation, but were insufficient on their own to account for the full between-phenotype risk gradient.

**TABLE 4:** Baseline characteristics of the MIMIC 8-h complete-case derivation cohort by phenotype.

Variable	Overall	Phenotype 1	Phenotype 2	Phenotype 3	Phenotype 4
Patients, *n*	2,671	606	652	961	452
Age, years, mean (SD)	65.84 (15.79)	67.59 (15.02)	62.60 (16.05)	67.87 (14.79)	63.87 (17.45)
Female sex, %	41.33	42.90	39.11	39.44	46.46
Pre-landmark SOFA, mean (SD)	4.95 (2.57)	5.36 (2.86)	4.90 (2.55)	5.00 (2.51)	4.36 (2.16)
Baseline lactate, mmol/L, mean (SD)	4.76 (3.27)	5.49 (3.92)	4.50 (3.08)	4.80 (3.12)	4.06 (2.66)

Continuous variables are shown as mean (SD). MAP deficit burden, lactate clearance, and standardized urine output were phenotype-defining variables and are therefore not repeated in this baseline table.

### In-hospital mortality in the derivation cohort

Phenotype 1 showed the strongest mortality signal in MIMIC (Table 5). Crude in-hospital mortality was 47.03% in Phenotype 1, compared with 28.68% in Phenotype 2, 29.24% in Phenotype 3, and 17.70% in Phenotype 4. After adjustment for age, sex, and pre-landmark SOFA, the association remained strongest for Phenotype 1 versus Phenotype 4 (OR 3.67, 95% CI 2.74–4.93; *P* < 0.001). Phenotype 2 and Phenotype 3 also remained associated with higher mortality relative to Phenotype 4, with adjusted ORs of 1.80 (95% CI 1.33–2.42) and 1.76 (95% CI 1.33–2.34), respectively. These findings indicate that the mortality gradient across phenotypes was not eliminated by adjustment for age, sex, and pre-landmark SOFA.

**TABLE 5:** In-hospital mortality and secondary renal outcomes by phenotype in MIMIC.

Phenotype	In-hospital mortality, *n*/*N* (%)	Adjusted OR (95% CI) for in-hospital mortality vs. Phenotype 4	72-h AKI onset/progression, *n*/*N* (%)*	72-h incident RRT, *n*/*N* (%)**
Phenotype 1	285/606 (47.03)	3.67 (2.74–4.93)	284/606 (46.86)	85/606 (14.03)
Phenotype 2	187/652 (28.68)	1.80 (1.33–2.42)	287/652 (44.02)	45/652 (6.90)
Phenotype 3	281/961 (29.24)	1.76 (1.33–2.34)	426/961 (44.33)	89/961 (9.26)
Phenotype 4	80/452 (17.70)	Reference	146/452 (32.30)	7/452 (1.55)

Adjusted mortality models included age, sex, and pre-landmark SOFA. For secondary renal outcomes, *n*/*N* (%) are reported using total phenotype counts as denominators. Accordingly, these renal outcomes are descriptive phenotype-level event summaries rather than formal risk-set incidence estimates. No adjusted effect models were estimated for the renal secondary outcomes. *AKI onset/progression was defined from the 8-h landmark using the prespecified creatinine + CRRT-based route. ** Incident RRT was assessed over 72 h from the 8-h landmark.

### Secondary renal outcomes

Secondary renal outcomes are reported as descriptive event summaries only (Table 5). Because body-weight-standardized urine output contributed directly to phenotype construction, these renal outcomes were not interpreted as independent validation of the phenotype structure. Using total phenotype counts as denominators in the archived summaries, the crude rate of 72-h AKI onset/progression was 46.86% in Phenotype 1, 44.02% in Phenotype 2, 44.33% in Phenotype 3, and 32.30% in Phenotype 4. The corresponding 72-h incident RRT proportions were 14.03, 6.90, 9.26, and 1.55%, respectively. These patterns are clinically consistent with the low-urine-output and perfusion-failure profile of Phenotype 1, but they should be viewed as expected descriptive renal event gradients rather than as independent or adjusted evidence supporting the phenotype construct.

### Stability, selection, and incremental-value analyses

Resampling analyses showed moderate-to-good but variable stability of the four-class structure (Supplementary Table 2). The median ARI was 0.862 (IQR 0.765–0.911) across 30 bootstrap repetitions and 0.879 (IQR 0.781–0.900) across 20 split-sample repetitions. These results support good central-tendency reproducibility of the retained four-class partition. Inspection of the recovered diagonal GMM parameters showed that the smallest recovered variance was for standardized MAP deficit burden in Phenotype 2 (variance = 0.042), indicating a relatively compact low-MAP-deficit component in this dimension. This feature did not eliminate overall partition reproducibility, as reflected by the bootstrap and split-sample ARI summaries, but it supports cautious interpretation of the exact class geometry, particularly for Phenotype 2.

Selection into the complete-case cohort was clearly non-random (Supplementary Table 3). Compared with excluded patients at the MIMIC landmark, included patients had higher baseline lactate (4.76 vs. 3.54 mmol/L), higher SOFA (4.95 vs. 4.49), and higher crude in-hospital mortality (31.19% vs. 19.38%). This pattern was clinically plausible, given that repeat lactate measurement is typically triggered by greater severity and ongoing concern rather than generated at random. IPW sensitivity analysis was used to examine whether measured differences in complete-case inclusion materially altered the mortality association (Supplementary Table 4). For Phenotype 1 versus Phenotype 4, the OR changed only modestly from 3.67 (95% CI 2.74–4.93) in the primary model to 3.54 (95% CI 2.81–4.47) after IPW. However, because repeat lactate is a clinically triggered reassessment variable, this sensitivity analysis cannot remove the intrinsic dependence of lactate clearance on illness severity and monitoring intensity.

An expanded adjusted sensitivity model in MIMIC, additionally incorporating broader pre-landmark case-mix covariates, attenuated effect estimates but preserved the overall mortality pattern (Supplementary Table 4). Relative to Phenotype 4, the adjusted OR was 2.56 (95% CI 1.85–3.53; *P* < 0.001) for Phenotype 1, 1.59 (95% CI 1.14–2.20; *P* = 0.006) for Phenotype 2, and 1.31 (95% CI 0.97–1.79; *P* = 0.083) for Phenotype 3. Thus, broader case-mix adjustment attenuated the phenotype-mortality associations, particularly for Phenotype 3, while the association for Phenotype 1 remained the strongest and statistically robust.

Incremental-value analyses clarified what the phenotype adds and what it does not (Supplementary Table 5). The clinical base model of age + sex + SOFA yielded an AUC of 0.608 and a Brier score of 0.207. Adding phenotype improved the AUC to 0.659 and the Brier score to 0.200, with a likelihood-ratio test *P* < 0.001. However, the phenotype did not provide the strongest predictive representation of the perfusion variables. Urine output alone yielded a higher AUC than phenotype membership (0.668 vs. 0.659), and direct modeling of the continuous triplet performed best overall, with an AUC of 0.685 and a Brier score of 0.194. Thus, although phenotype membership added prognostic information beyond a simple clinical base model, discretizing the three continuous perfusion variables into latent classes resulted in lower apparent predictive performance than both a single strong constituent variable and the continuous triplet. The phenotype should therefore not be interpreted as a performance-optimizing risk-prediction representation of these variables.

### Fixed-parameter external transportability assessment in eICU

Fixed-parameter external transportability assessment in eICU was performed by applying the MIMIC-derived preprocessing constants and recovered four-class diagonal GMM parameter set without external refitting (Supplementary Tables 6, 7). Because phenotype assignment required complete availability of MAP, urine output, and repeat lactate, both the strict and relaxed eICU transfer cohorts should be understood as measured subsets rather than the full operational cohorts. In eICU, the operational septic shock candidate cohort included 2,277 patients, from which 2,163 entered the strict 8-h landmark cohort and 2,090 entered the relaxed 12-h landmark cohort (Tables 2A,B). Overall crude in-hospital mortality in the complete-case eICU cohorts was 34.26% in the strict structure and 33.33% in the relaxed structure.

Phenotype contour and crude risk layering were partially recoverable under fixed-parameter transfer (Table 6). In the primary strict 8-h transportability analysis, Phenotype 1 accounted for 174/432 patients (40.28%), had a mean maximum posterior probability of 0.867, and showed the highest in-hospital mortality (44.25%) and ICU mortality (35.06%). In the prespecified relaxed 12-h sensitivity transportability analysis, Phenotype 1 accounted for 276/666 patients (41.44%), had a mean maximum posterior probability of 0.876, and again showed the highest in-hospital mortality (43.48%) and ICU mortality (36.59%). However, assignment certainty was not uniform across transferred classes. Phenotype 1 and Phenotype 3 had lower mean maximum posterior probabilities than Phenotype 2 and Phenotype 4, whereas the rare Phenotype 2 group showed near-perfect assignment despite very small sample size. Thus, the eICU results support recovery of a high-risk perfusion contour and crude risk layering, but not equally robust transfer of all individual latent classes. In both structures, Phenotype 4 remained the lowest-risk phenotype, with in-hospital mortality below 20% and ICU mortality near 13%.

**TABLE 6:** Fixed-parameter external transportability results in eICU under strict and relaxed transfer structures.

Cohort	Phenotype	Patients, *n* (%)	Mean maximum posterior probability	In-hospital mortality, %	ICU mortality, %
Strict 8 h	Phenotype 1	174 (40.28)	0.867359	44.25	35.06
Strict 8 h	Phenotype 2	18 (4.17)	0.988577	22.22	11.11
Strict 8 h	Phenotype 3	139 (32.18)	0.841602	33.81	24.46
Strict 8 h	Phenotype 4	101 (23.38)	0.933858	19.80	12.87
Relaxed 12 h	Phenotype 1	276 (41.44)	0.876312	43.48	36.59
Relaxed 12 h	Phenotype 2	28 (4.20)	0.990099	21.43	14.29
Relaxed 12 h	Phenotype 3	214 (32.13)	0.835426	31.31	23.36
Relaxed 12 h	Phenotype 4	148 (22.22)	0.920368	19.59	12.84

Assignment used fixed MIMIC-derived preprocessing constants and recovered four-class diagonal GMM parameters without external refitting. Phenotype 1 remained the highest-risk class in both transfer structures, but class-prevalence drift was evident relative to the derivation cohort. Mean maximum posterior probabilities should be interpreted alongside class size: Phenotype 1 and Phenotype 3 had lower assignment certainty than the sparse Phenotype 2 group, whose near-perfect posterior probability should not be interpreted as stronger external robustness.

At the same time, the eICU transportability assessment revealed notable class-prevalence drift (Table 6). Relative to the derivation cohort, Phenotype 1 was substantially more common in eICU, whereas Phenotype 2 became rare (4.17% in the strict structure and 4.20% in the relaxed structure). This drift should be interpreted descriptively rather than as evidence of true between-database differences in phenotype prevalence. Because eICU used a diagnosis-anchored entry strategy and frozen assignment was restricted to patients with complete MAP, urine output, and repeat-lactate information, the observed class distribution cannot be separated from differences in cohort entry definitions and measurement-dependent sample formation. Included-versus-excluded analyses further showed that complete-case transfer was strongly shaped by measurement availability: among excluded patients, MAP was almost always recoverable, whereas urine output and repeat lactate were far less frequently available (Supplementary Tables 8, 9). Notably, the selection direction differed from that observed in MIMIC: eICU excluded patients had slightly higher crude in-hospital mortality than included patients in both the strict and relaxed structures. This pattern may reflect early death, rapid deterioration, or other clinical trajectories occurring before repeat lactate or complete urine-output reassessment could be captured, and it may have attenuated the apparent severity of the high-risk contour in the measured complete-case transfer subsets. Taken together, these findings support detectability of the high-risk contour and overall crude risk layering under fixed-parameter transfer, while interpretation is constrained by non-equivalent cohort construction, class-prevalence drift, and marked measurement-dependent sample formation. Accordingly, external interpretation should center on recovery of the Phenotype 1 high-risk contour among measured eICU subsets rather than on replication of class prevalence or of every individual latent class.

## Discussion

In this landmark-defined study of septic shock, we identified four early perfusion-related phenotypes using three routinely available bedside variables: MAP deficit burden, lactate clearance, and body-weight-standardized urine output. One phenotype, Phenotype 1, consistently represented the highest-risk contour, characterized by greater hemodynamic deficit, poorer lactate clearance, and lower urine output. This risk gradient persisted after adjustment for age, sex, and pre-landmark SOFA. Secondary renal event summaries followed expected descriptive gradients, but were not treated as independent validation because body-weight-standardized urine output contributed to phenotype construction. Under frozen transfer to eICU, the high-risk contour and crude risk layering of Phenotype 1 remained detectable in both strict and relaxed structures, although not all transferred classes were reproduced with comparable prevalence.

The attenuation observed in the expanded adjustment model indicates that part of the phenotype-mortality association was explained by measurable pre-landmark case mix, especially for the weaker non-reference phenotypes; therefore, the primary adjusted estimates should be interpreted as parsimonious association estimates rather than fully adjusted causal or prognostic effects.

The lactate-clearance component also requires cautious interpretation. Repeat lactate availability and the resulting clearance value are not generated independently of clinical status; they reflect a monitoring and reassessment process triggered by severity, ongoing resuscitation, and clinician concern. Accordingly, the phenotype should be interpreted as an early monitored perfusion-state descriptor in patients with complete reassessment data. It partly captures the intersection of illness severity, monitoring intensity, and failure of early physiologic improvement, rather than a latent biological subtype separable from clinical measurement processes.

A central contribution of the present study is its deliberate focus on a narrow and clinically coherent perfusion domain. Prior sepsis subphenotyping work has identified meaningful patient groups using broader combinations of clinical variables and latent-class approaches (6, 7), as well as transcriptomic, trajectory-based, and representation-learning frameworks (23–27). Those frameworks have advanced the field substantially, but they often span multiple biologic or syndromic domains at once. By contrast, our approach asked a more specific question: whether a clinically interpretable early perfusion state could be captured using three routine bedside measures that clinicians already interpret during early resuscitation. In that sense, Phenotype 1 should be viewed less as a generic “severe sepsis” class and more as a persistent early perfusion-dysfunction state.

The study also addresses a methodological problem common to observational phenotype research: temporal misalignment between phenotype construction and outcome follow-up (14, 15). Because repeat lactate is not available at ICU admission and is typically obtained in response to ongoing clinical concern, analyses that use later reassessment data to define phenotypes while initiating follow-up at time zero are vulnerable to exposure–risk misalignment and immortal-time-type bias (14, 15). By requiring that all phenotype-defining information be observed before follow-up began, our 8-h landmark design mitigated this problem and provided a more temporally coherent framework for interpreting phenotype–outcome associations. At the same time, this design choice improves interpretability rather than establishing causal inference.

The fixed-parameter eICU transportability results deserve a deliberately qualified interpretation. We did not refit the model in eICU; instead, we transferred the MIMIC-derived preprocessing constants and recovered four-class diagonal GMM parameters. This design tested transferability of phenotype contours rather than allowing the external cohort to derive a new latent structure. Under this fixed-parameter design, Phenotype 1 remained the highest-risk class in both strict and relaxed structures, whereas Phenotype 4 remained the lowest-risk class, supporting detectability of the high-risk contour and overall crude risk layering. At the same time, class-prevalence drift was substantial, with Phenotype 1 more common and Phenotype 2 markedly rarer in eICU than in MIMIC. However, this drift should not be interpreted as a true epidemiologic shift in phenotype prevalence across databases, because entry definitions and measurement-density filters differed simultaneously. Equally important, phenotype assignment in eICU was feasible only in measured subsets of the operational cohorts, because urine output and repeat-lactate availability, rather than MAP availability, were the principal determinants of complete-case formation. Not all transferred classes appeared equally robust in eICU, and the externally infrequent Phenotype 2 should therefore be interpreted cautiously; the eICU findings are better viewed as evidence that a high-risk perfusion-failure contour can be recovered under fixed-parameter transfer than as full independent validation of every latent partition. The posterior-probability pattern further supports this caution: Phenotype 1 and Phenotype 3 were assigned with lower certainty than the sparse Phenotype 2 group, whose near-perfect posterior probability may reflect the behavior of frozen GMM boundaries in a small externally mapped class rather than robust replication of that class. Accordingly, the eICU findings should not be generalized to the entire eICU operational septic shock cohort. They indicate that the MIMIC-derived high-risk perfusion contour was recoverable among patients with sufficient early monitoring density for all three phenotype-defining variables, rather than demonstrating transportability to septic shock patients irrespective of measurement availability.

The selection and incremental-value analyses further clarify the interpretive scope of this phenotype framework. Complete-case inclusion in MIMIC was clearly non-random, with included patients showing higher baseline lactate, higher SOFA, and higher crude mortality than excluded patients. However, IPW sensitivity analysis did not materially alter the primary mortality association, suggesting that non-random selection is more likely to constrain generalizability than to overturn the main mortality signal. At the same time, the incremental-value analysis showed that phenotype membership improved apparent performance beyond a simple clinical base model, but did not outperform urine output alone and remained inferior to direct modeling of the three continuous phenotype-defining variables. This finding indicates that discretization into four classes incurred information loss relative to the original continuous perfusion signals. The phenotype framework should therefore not be interpreted as a superior individual-level prediction model. Its intended role is narrower: to provide a clinically interpretable subgroup structure that may support communication, mechanistic hypothesis generation, and possible trial enrichment.

These findings should also be interpreted in the context of the dynamic nature of sepsis heterogeneity. Recent studies suggest that clinically derived sepsis subtypes may evolve over time rather than remain fixed from presentation onward (24–26, 28). Our phenotypes should therefore be understood as early-state descriptors with prognostic relevance, not immutable patient identities. This interpretation is consistent with both the landmark design and the physiologic construction of our model: the phenotype reflects a patient’s early perfusion state within a defined window, not a fixed biologic subtype.

Several limitations merit emphasis. First, this was a retrospective observational analysis, and no treatment-effect, treatment-response, or mediation claims can be made. The primary outcome was in-hospital mortality rather than fixed-time mortality. Although this endpoint was selected to maintain consistent outcome ascertainment across MIMIC-IV and eICU, it may be influenced by discharge timing, interfacility transfer practices, palliative-care transitions, bed availability, and local healthcare-system factors. If these practices differ across phenotypes or databases, absolute mortality rates and estimated phenotype-mortality associations could be biased. Therefore, future studies with harmonized post-discharge vital-status follow-up should evaluate fixed-time outcomes such as 28-day or 90-day mortality. Second, derivation relied on a complete-case cohort, and lactate clearance was intrinsically linked to illness severity and clinical monitoring because repeat lactate acquisition was clinically triggered rather than random. Although IPW sensitivity analysis addressed measured differences in complete-case inclusion, it cannot remove the endogeneity of lactate clearance itself. Therefore, the high-risk phenotype should be interpreted as a monitored early perfusion-state pattern rather than as evidence of a latent class independent of measurement processes. Third, case identification was harmonized but not identical across databases, with full Sepsis-3 reconstruction in MIMIC and a diagnosis-anchored entry strategy in eICU. Fourth, the retained diagonal GMM was selected for interpretability and transferability rather than as a definitive generative model. MAP deficit burden and urine output are zero-inflated or skewed bedside variables, and the smallest recovered diagonal variance was observed for standardized MAP deficit burden in Phenotype 2 (variance = 0.042). Although the median resampling ARIs supported good central-tendency reproducibility, the compact Phenotype 2 MAP-deficit component still supports cautious interpretation of the exact diagonal-GMM class geometry; therefore, future analyses should examine constrained or regularized mixture models, latent-class approaches with distribution-specific assumptions, rank-based clustering, non-Gaussian mixture models, or non-parametric clustering alternatives. In addition, the selected number of classes should be viewed as a pragmatic reporting choice rather than an objectively identified true number of latent states, because information criteria continued to improve with higher-class solutions and interpretability-based judgment was required. Fifth, renal outcomes were secondary and descriptive. Because body-weight-standardized urine output was a phenotype-defining variable, higher AKI and RRT proportions in low-urine-output phenotypes are partly construct-related and physiologically expected rather than independent corroborating evidence. In addition, renal outcomes were summarized using archived phenotype-level denominators rather than formal risk-set denominators, adjusted models, or time-to-event analyses. Sixth, fixed-parameter phenotype assignment in eICU was limited to measured subsets of the operational cohorts, because complete-case formation depended largely on urine output and repeat-lactate availability; this constrains generalizability beyond patients with comparable early measurement density. No eICU weighting analysis was performed to reweight the frozen-assignment subset back to the full operational cohort; therefore, the external transportability findings should be interpreted as subset-based contour recovery among patients with sufficient early measurement density. Although missing eligible weight documentation did not exclude otherwise complete patients from phenotype assignment in either database, body-weight-standardized urine output remains dependent on the accuracy and timing of ICU weight documentation. Estimated or non-contemporaneous weights may still introduce measurement error in standardized urine-output estimates. Finally, our intentionally parsimonious framework prioritizes bedside feasibility over comprehensive biologic representation. Whether linkage with inflammatory, endothelial, transcriptomic, or treatment-response signatures can improve biologic interpretability without sacrificing clinical usability remains an important question for future work (29).

Overall, the present findings support a restrained interpretation. A phenotype defined by greater cumulative hypotensive burden, poorer early lactate resolution, and reduced urine output identifies a subgroup of septic shock patients with consistently higher crude and adjusted mortality in MIMIC and a recoverable high-risk contour under frozen eICU transfer. The framework should be considered a hypothesis-generating, interpretable prognostic stratification approach for patients with sufficient early monitoring density, not a validated clinical decision tool.

## Conclusion

Within a strict 8-h landmark framework, early perfusion phenotyping identified a high-risk contour in septic shock defined by greater MAP deficit burden, poorer lactate clearance, and lower urine output. Under fixed-parameter external transportability assessment in eICU, this high-risk contour remained detectable in measured complete-case subsets, but the small and highly selected external cohorts, class-prevalence drift, and measurement-dependent sample formation preclude robust conclusions about transportability of the entire four-class model, particularly for Phenotypes 2–4. Early perfusion phenotyping may provide a clinically interpretable approach to subgroup description and prognostic stratification, but it should not be viewed as improving individual-level prediction beyond direct modeling of the underlying continuous perfusion variables.

## Data Availability

Publicly available datasets were analyzed in this study. The datasets analyzed in this study are publicly available through PhysioNet: MIMIC-IV: Medical Information Mart for Intensive Care IV, PhysioNet. Available at: https://physionet.org/content/mimiciv/; eICU Collaborative Research Database: eICU Collaborative Research Database v2.0, PhysioNet. Available at: https://physionet.org/content/eicu-crd/2.0/. No accession numbers apply. Access to both datasets requires PhysioNet credentialing, completion of the required human-subjects research training, and agreement to the applicable data use terms. MIMIC-IV and eICU are de-identified critical care databases made available to credentialed users through PhysioNet.
