# Supplementary material for: Landmark-based early perfusion phenotyping for prognostic stratification in septic shock: derivation in MIMIC-IV and fixed-parameter external transportability assessment in eICU
Source: Front Med (Lausanne). 2026 Jul 28;13:1880921. doi: 10.3389/fmed.2026.1880921 (PMC13457294; doi:10.3389/fmed.2026.1880921)
Supplement: Supplementary file 1 [file Table_1.docx]

**Landmark-based early perfusion phenotyping for prognostic stratification in septic shock: derivation in MIMIC-IV and fixed-parameter external transportability assessment in eICU**

Authors: Shuo Yang, Zhihao Ren, Yiran Li, Zihang Zhao, Changsheng Sun, Lin Liu, Lin Jin, Ling Wang, Zhiyong Hou

Supplementary appendix containing Supplementary Tables S1-S9 and supporting methodological details

**Supplementary Appendix**

The supplementary tables provide supporting evidence for class-number selection, phenotype stability, complete-case selection patterns, IPW sensitivity analysis, incremental-value analysis, and fixed-parameter external transportability assessment. These materials are intended to complement the main manuscript and should be interpreted in conjunction with the landmark-based study design and the deliberately limited external transportability scope.

# **Supplementary Tables**

**Supplementary Table S1. Multi-criterion numerical comparison of 2–6 diagonal GMM solutions in MIMIC**

| **No. of classes** | **AIC** | **BIC** | **Minimum class proportion, %** | **Mean maximum posterior probability** | **Bootstrap ARI, median**  **(IQR)** | **Split-sample ARI, median**  **(IQR)** | **Interpretive summary** | **Decision** |
| --- | --- | --- | --- | --- | --- | --- | --- | --- |
| 2 | 19625.155 | 19701.727 | 37.81 | 0.920871 | 0.923 (0.868–0.955) | 0.916 (0.822–0.944) | Stable but overly coarse partition; key feature-profile separation was merged | Not retained |
| 3 | 18161.935 | 18279.739 | 18.98 | 0.874108 | 0.328 (0.225–0.587) | 0.516 (0.271–0.801) | Less stable partition with insufficient separation of the high-risk contour | Not retained |
| 4 | 13375.343 | 13534.379 | 16.92 | 0.896021 | 0.862 (0.765–0.911) | 0.879 (0.781–0.900) | Best pragmatic balance of feature-profile separation, class size, assignment certainty, stability, and interpretability | Retained |
| 5 | 13231.666 | 13431.933 | 4.19 | 0.887145 | 0.612 (0.566–0.688) | 0.661 (0.602–0.729) | Further improvement in information criteria but fragmented small class and weaker stability | Not retained |
| 6 | 12713.173 | 12954.671 | 5.39 | 0.847791 | 0.649 (0.570–0.745) | 0.689 (0.566–0.852) | Lowest information criteria but lower assignment certainty, more fragmented classes, and weaker stability | Not retained |

Note: Candidate solutions were compared using a multi-criterion framework that considered AIC, BIC, minimum class proportion, mean maximum posterior probability, bootstrap stability, split-sample stability, and clinical interpretability of the resulting feature profiles. Stability was summarized using the adjusted Rand index (ARI). Because AIC and BIC continued to improve with higher-class solutions, the retained four-class solution should not be interpreted as the objectively optimal density model or as proof of a unique true number of latent states. The four-class solution was retained as a pragmatic parsimonious representation that balanced class separation, class size, assignment certainty, stability, and interpretability while avoiding increasingly fragmented higher-class solutions.

For resampling stability, each bootstrap or split-sample model was refitted and then used to assign all 2,671 complete-case patients; ARI was calculated against the corresponding full-sample solution with the same number of classes.

**Supplementary Table S2. Phenotype stability analyses in MIMIC**

| **Analysis** | **No. of repetitions** | **Median ARI** | **IQR** |
| --- | --- | --- | --- |
| Bootstrap | 30 | 0.862 | 0.765–0.911 |
| Split-sample | 20 | 0.879 | 0.781–0.900 |

Note: Stability was summarized using the adjusted Rand index (ARI), comparing repeated resampling-based solutions with the corresponding full-sample four-class solution. Dispersion is reported as the interquartile range (IQR). These results support good central-tendency reproducibility of the retained four-class solution, although the solution should still be interpreted as a pragmatic working partition rather than as a definitive generative density model.

**Supplementary Table S3. Included versus excluded patients at the MIMIC 8-h landmark: complete-case selection analysis**

| **Variable** | **Included complete-case (n=2,671)** | **Excluded (n=1,703)** | **Standardized mean difference** |
| --- | --- | --- | --- |
| Age, mean | 65.84 | 67.14 | -0.085 |
| Female sex, % | 41.33 | 40.11 | 0.025 |
| Pre-landmark SOFA, mean | 4.95 | 4.49 | 0.185 |
| Baseline lactate, mean | 4.76 | 3.54 | 0.431 |
| In-hospital mortality, % | 31.19 | 19.38 | 0.272 |

Note: Included patients had complete availability of all three phenotype-defining features at the 8-h landmark; excluded patients lacked at least one phenotype-defining feature. Standardized mean differences are presented descriptively.

**Supplementary Table S4. Primary adjusted, expanded adjusted, and IPW in-hospital mortality models in MIMIC**

| **Model** | **Comparison** | **OR (95% CI) for in-hospital mortality** | **P value** |
| --- | --- | --- | --- |
| Primary adjusted model | Phenotype 2 vs Phenotype 4 | 1.80 (1.33-2.42) | <0.001 |
| Primary adjusted model | Phenotype 3 vs Phenotype 4 | 1.76 (1.33-2.34) | <0.001 |
| Primary adjusted model | Phenotype 1 vs Phenotype 4 | 3.67 (2.74-4.93) | <0.001 |
| Expanded adjusted model | Phenotype 2 vs Phenotype 4 | 1.59 (1.14-2.20) | 0.006 |
| Expanded adjusted model | Phenotype 3 vs Phenotype 4 | 1.31 (0.97-1.79) | 0.083 |
| Expanded adjusted model | Phenotype 1 vs Phenotype 4 | 2.56 (1.85-3.53) | <0.001 |
| IPW model | Phenotype 2 vs Phenotype 4 | 1.76 (1.39-2.23) | <0.001 |
| IPW model | Phenotype 3 vs Phenotype 4 | 1.69 (1.35-2.11) | <0.001 |
| IPW model | Phenotype 1 vs Phenotype 4 | 3.54 (2.81-4.47) | <0.001 |

Note: The primary adjusted model included age, sex, and pre-landmark SOFA. The expanded adjusted model additionally included baseline lactate, grouped first ICU care unit, pre-landmark invasive ventilation status, and pre-landmark RRT. Phenotype-defining variables were not re-entered into the expanded model. IPW was used as a sensitivity analysis to address non-random selection into the complete-case derivation cohort.

**Supplementary Table S5. Incremental value analysis for in-hospital mortality in MIMIC**

| **Model** | **AUC** | **Brier score** | **ΔAUC vs clinical base model** | **Likelihood-ratio test P value vs clinical base model** |
| --- | --- | --- | --- | --- |
| Clinical base model (age + sex + SOFA) | 0.608 | 0.207 | - | - |
| Base model + MAP deficit burden | 0.635 | 0.202 | 0.027 | <0.001 |
| Base model + lactate clearance | 0.635 | 0.204 | 0.027 | <0.001 |
| Base model + urine output | 0.668 | 0.199 | 0.059 | <0.001 |
| Base model + phenotype | 0.659 | 0.200 | 0.051 | <0.001 |
| Base model + continuous triplet | 0.685 | 0.194 | 0.077 | <0.001 |

Note: The phenotype improved discrimination beyond the clinical base model, but apparent predictive performance remained better when the continuous triplet was modeled directly. The continuous triplet model represents direct modeling of the three phenotype-defining variables without discretization into latent classes.

**Supplementary Table S6. Fixed winsorization cut points and standardization constants transferred from MIMIC to eICU**

| **Parameter** | **Value** | **Parameter** | **Value** |
| --- | --- | --- | --- |
| p01_map | 0 | p99_map | 13.543479 |
| p01_uo | 0 | p99_uo | 4.975305 |
| p01_lac | -138.666667 | p99_lac | 75.214286 |
| mean_map_w | 1.728760 | sd_map_w | 2.525489 |
| mean_uo_w | 0.909621 | sd_uo_w | 0.987780 |
| mean_lac_w | 7.422104 | sd_lac_w | 39.882760 |

Note: map, lac, and uo denote MAP deficit burden, lactate clearance, and urine output, respectively. p01 and p99 denote the 1st and 99th percentile winsorization cut points; mean_*_w and sd_*_w denote the post-winsorization means and standard deviations used for standardization.

**Supplementary Table S7. Recovered 4-class diagonal GMM parameters used for fixed-parameter transfer**

| **Internal class ID** | **Manuscript phenotype** | **Weight** | **Mean vector (map, lac, uo)** | **Variance vector (map, lac, uo)** |
| --- | --- | --- | --- | --- |
| 0 | Phenotype 2 | 0.242040 | (-0.684484, 0.135523, 0.178660) | (0.042038, 0.809710, 1.144306) |
| 1 | Phenotype 3 | 0.322090 | (-0.377246, 0.215215, -0.353683) | (0.054127, 0.495604, 0.171112) |
| 2 | Phenotype 1 | 0.235414 | (1.122348, -0.486477, -0.595792) | (1.763845, 1.416039, 0.077161) |
| 3 | Phenotype 4 | 0.200455 | (0.114555, 0.061874, 1.052272) | (0.541495, 1.171409, 1.475839) |

Note: Mean and variance vectors are ordered as (z-map, z-lac, z-uo), corresponding to standardized MAP deficit burden, lactate clearance, and urine output. These recovered parameters were applied directly during fixed-parameter transfer to eICU without external refitting.

Additional modeling caveat: The smallest recovered diagonal variance was observed for z-MAP in Phenotype 2 (variance = 0.042), indicating a relatively compact low-MAP-deficit component in the fitted diagonal GMM. This should be interpreted alongside the resampling stability summaries in Supplementary Table S2. Although this compact component did not eliminate overall partition reproducibility, it supports interpreting the retained solution as a clinically interpretable stratification framework rather than as a definitive generative density model. Future analyses should evaluate constrained or regularized mixture models, latent-class approaches with distribution-specific assumptions, rank-based clustering, non-Gaussian mixture models, or non-parametric clustering approaches that are less sensitive to zero-inflated and skewed bedside variables.

**Supplementary Table S8. Included versus excluded patients in eICU under the strict 8-h transfer structure**

| **Group** | **Patients, n** | **Mean age, years** | **Female, %** | **Baseline lactate, mmol/L** | **In-hospital mortality, %** | **ICU mortality, %** |
| --- | --- | --- | --- | --- | --- | --- |
| Included | 432 | 64.83 | 44.68 | 5.86 | 34.26 | 25.46 |
| Excluded | 1,731 | 67.50 | 45.98 | 5.25 | 36.34 | 28.77 |

Note: Included patients had complete availability of MAP, urine output, and lactate by construction. Among excluded patients, MAP availability was 99.36%, urine output availability was 35.93%, and lactate availability was 23.97%.

**Supplementary Table S9. Included versus excluded patients in eICU under the relaxed 12-h transfer structure**

| **Group** | **Patients, n** | **Mean age, years** | **Female, %** | **Baseline lactate, mmol/L** | **In-hospital mortality, %** | **ICU mortality, %** |
| --- | --- | --- | --- | --- | --- | --- |
| Included | 666 | 65.35 | 42.64 | 5.54 | 33.33 | 26.13 |
| Excluded | 1,424 | 67.53 | 46.98 | 5.14 | 34.69 | 26.83 |

Note: Included patients had complete availability of MAP, urine output, and lactate by construction. Among excluded patients, MAP availability was 99.23%, urine output availability was 25.35%, and lactate availability was 41.99%.
